# Synthesis and characterization of TiO_2_-based supported materials for industrial application and recovery in a pilot photocatalytic plant using chemometric approach

**DOI:** 10.1007/s11356-024-32467-y

**Published:** 2024-02-20

**Authors:** Nicolò Ghibaudo, Maurizio Ferretti, Entesar Al-Hetlani, Metwally Madkour, Mohamed O. Amin, Stefano Alberti

**Affiliations:** 1 Chemistry and Industrial Chemistry Department, University of Genoa, Via Dodecaneso 31, 16146 Genoa (Ge), Italy; 2 Chemistry Department, Faculty of Science, Kuwait University, P.O. Box 5969, 13060 Safat, Kuwait; 3 Chemistry Department, Faculty of Science, Arish University, Al-Arish, 45511 Egypt

**Keywords:** Heterogeneous photocatalysis, Chemometrics, Point of zero charge, Pilot plant, Enhanced sedimentation

## Abstract

In this contribution, the performance of powdered titanium dioxide (TiO_2_)–based photocatalysts was evaluated in a pilot photocatalytic plant for the degradation of different dyes, with an investigated volume of 1 L and solar simulated light as irradiation source. Five different samples, synthesized in our laboratories, were tested in the pilot plant, each consisting of TiO_2_ nanoparticles (NPs) coupled with a different material (persistent luminescent material and semiconductor material) and treated in different thermal conditions. All synthesized samples have been subjected to X-ray diffraction (XRD), X-ray photoelectron spectroscopy (XPS), Brunauer–Emmett–Teller analysis (BET), and transmission electron microscopy (TEM) characterization, to shed light on the influence of introducing other materials on titania characteristics. To study and evaluate the significance of the parameters affecting the process in the pilot plant, a chemometric approach was applied, by selecting a mathematical model (D-Optimal) to simultaneously monitor a large number of variables (i.e., 7), both qualitative and quantitative, over a wide range of levels. At the same time, the recovery of the synthesized photocatalysts was studied following a novel promising recuperation method, i.e., annulling the surface charge of the suspended samples by reaching the isoelectric point (pH_PZC_) of each sample, for the quantitative precipitation of TiO_2_ nanoparticles.

**Graphical Abstract:**

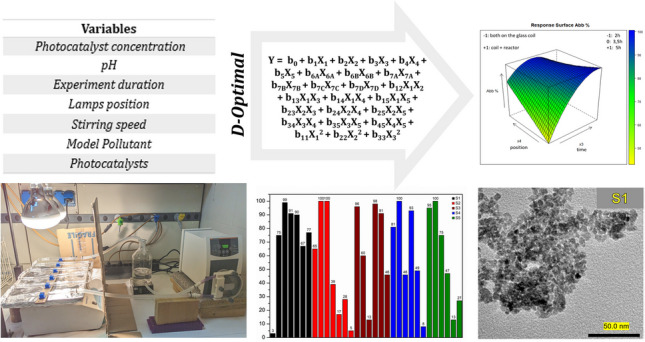

**Supplementary Information:**

The online version contains supplementary material available at 10.1007/s11356-024-32467-y.

## Introduction

Water is undoubtedly a vital natural resource for mankind and for all ecosystems, and its preservation from the increasing consumption represents one of the main environmental challenges of the current century (Sousa et al. 2018). In addition, the constant replenishment of numerous chemical substances in water bodies is posing additional threats: These compounds, which have the potential to cause hostile effects on the environment and human health, are often collectively referred to as emerging contaminants (ECs) (Pal et al. 2010; Rykowska and Wasiak 2015). They include a wide range of different compounds, together with their degradation by-products, such as pharmaceuticals, personal care products, pesticides, veterinary products, and industrial additives (Alberti et al. 2021c; Yang et al. 2017). Such contaminants are characterized by a wide range of physical–chemical properties and very low concentrations (Ahmed et al. 2021; Dulio et al. 2018). Among the treatments designed for the removal of emerging pollutants, advanced oxidation processes (AOPs) are considered the most promising approach (Abdulrazaq et al. 2021; Deng and Zhao 2015; Dulio et al. 2018; Koumaki et al. 2015); an example is represented by heterogeneous photocatalysis, which is extensively studied to deal with ECs and their respective reaction intermediates (Ribeiro et al. 2015). Photocatalysis represents a unique class of reactions, where a semiconductor material, the photocatalyst, upon absorption of photons with a proper wavelength, exploits the energy absorbed from the light to carry out chemical reactions (Antonopoulou et al. 2021; Yang and Wang 2018). The photocatalytic process has several advantages, such as complete mineralization of pollutants, no chemical consumption, absence of waste disposal problem, low cost, and mild temperature and pressure conditions (Bhatkhande et al. 2002; Rueda-Marquez et al. 2020; Yang and Wang 2018; Younis and Kim 2020).

The number of publications dealing with “photocatalysis” or “photocatalyst” (according to Scopus) is nonetheless raising (Rueda-Marquez et al. 2020) due to the many weaknesses that still affect the photocatalytic process, including maximization of light absorption, shift to the visible region, minimization of charge carriers recombination, maximization of charge transfer at the interface, avoidance of nanoparticle dispersion, and enhancement of recovery. Many approaches were performed to investigate how to overcome these issues, like doping, coupling with co-catalysts, and using different materials as support (Alberti et al. 2019, 2021b; Antonopoulou et al. 2021; Bhatkhande et al. 2002; Liu et al. 2021; Martinelli et al. 2018; Rueda-Marquez et al. 2020; Sivaraman et al. 2022; Tayyab et al. 2022, 2023; 2024; Yang and Wang 2018; Younis and Kim 2020). Among materials proposed for water decontamination, semiconductors like zinc oxide (ZnO) and TiO_2_ represent the most popular options. A great advantage is represented by the synthetic process, as TiO_2_ can be easily obtained through a soft chemistry synthetic route, e.g., sol–gel technique, which exploits mild temperature and pressure conditions and consequently a very low energy consumption. Furthermore, this synthetic approach allows an easy coupling with different types of innovative materials to endow the composite sample with additional features (Azeez et al. 2018; Bhatkhande et al. 2002; Sivaraman et al. 2022; Younis and Kim 2020). In this regard, it is has been recently documented how the photocatalyst supported on persistent luminescence materials (PeL) increased the overall efficiency and allowed the photocatalytic process to occur in the dark (Alberti et al. 2023; Azeez et al. 2018; Villa et al. 2016), which is significant in terms of reduction of energy consumption (Koumaki et al. 2015).

One of the major drawbacks related to the industrial use of photocatalysis is the difficulty to separate and recover the suspended photocatalyst after completing the water treatment process (Al-Hetlani et al. 2017). Different methods have already been proposed, such as the use of coagulants, ultrasonic radiation, or microfiltration, but they are expensive, often requiring special processes and/or the consumption of chemicals which may not be available to everyone. Nevertheless, there is still a strong need to establish alternative and simpler methods that can be appropriate for all types of nanosized photocatalysts (Umh and Kim 2014). One feasible example is represented by tailoring the surface charge of photocatalysts by adjusting the pH of the solution to reach the isoelectric point (pH_IEP_), or point of zero charge (pH_PZC_), where nanoparticles’ suspension becomes unstable due to the lack of electrostatic repulsion; hence, a rapid sedimentation of the photocatalyst occurs. In particular, pH_PZC_ is the pH value at which the surface charge of the NPs is equal to zero, while above and below the pH_PZC_, the nanoparticles possess a surface charge, which creates repulsion and maintain their colloidal stability as a suspension. Once the nanoparticles sedimented they can be easily separated, washed, and recycled again for more photocatalytic experiments. This should greatly reduce the amount of nanoparticles produced and consumed for future photocatalytic experiments (Al-Hetlani et al. 2017; Azeez et al. 2018).

In the present work, some of the major drawbacks related to the exploitation of heterogeneous photocatalysis were addressed and discussed. The prepared TiO_2_-based nanoparticles were characterized by means of several physico-chemical techniques (XRD, XPS, BET, TEM) to retrieve information on the influence of the coupling materials on the semiconductor. A photocatalytic pilot plant was set up, to simulate an industrial process exploiting heterogeneous photocatalysis for the degradation of emerging contaminants. A chemometric approach was applied to the photocatalytic tests performed with the pilot plant, to mathematically find out which significant variables should be controlled; these tests also included the assessment on the efficiency of five different synthetic photocatalysts. Eventually, an innovative recovery technique was tested with investigated samples, in order find out their pH_ZPC_, to avoid their dispersion and consequently allow their reuse in such pilot plants. To the best of our knowledge, the application of a chemometric approach to a photocatalytic pilot plant has never been reported so far, while the number of papers dealing with chemometrics for photocatalytic experiments is still very limited and only dealing with a small number of variables (Ba-Abbad et al. 2017; Boutra et al. 2022; Deriase et al. 2021).

## Materials and methods

### Chemicals

All descriptions of used reagents and materials are reported in SI.

### Photocatalyst synthesis

In the present work, five different TiO_2_-based photocatalysts were synthesized and investigated, and their synthetic conditions are summarized in Table 1 and reported below.

**Table 1:** Summary conditions of synthesized samples

*Sample*	*Composition*	*Synthesis*	*Thermal treatment conditions*
S1	TiO_2_	Gel (1:34:5)*	Hydrothermal treatment (3 h, 150 °C) Oven drying (24 h, 105 °C)
S2	TiO_2_/PeL	Gel (1:34:5)* 75% TiO_2_ gel + 0.5 g PeL	Hydrothermal treatment (6 h, 100 °C) Oven drying (24 h, 105 °C)
S3	TiO_2_	Gel (1:2:5)*	Oven drying (24 h, 105 °C) Solid-state treatment (1 h, 350 °C)
S4	TiO_2_/PeL	Gel (1:2:5)* TiO_2_/Pel (1:1)^#^	
S5	TiO_2_/ZnO/PeL	Gel (1:2:5)* TiO_2_/ZnO/PeL (1:1:2)^#^	

^*^(V/V)

^#^(w/w)

Each sample containing TiO_2_ was prepared according to the following synthetic procedure: The sol–gel process was performed using TTIP, i-PrOH and H_2_O in two different volume ratios, 1:2:5 and 1:34:5, respectively. TTIP and i-PrOH were mixed in an Erlenmeyer flask, to get a homogeneous solution. After a short time, water was quantitatively added and instantly the formation of the sol occurred. The solution was left under magnetic stirring for 4 h, to trigger the formation of the amorphous TiO_2_ gel, for both volume ratios. Subsequently, the crystallization of TiO_2_ was performed according to two different thermal treatments: a hydrothermal synthesis, carried out for 3 h at 150 °C in a Teflon reactor, filled to the half of the volume with the gel and immersed in a silicone oil bath; and a solid-state treatment, performed in a muffle furnace for 1 h at 350 °C on the powdered TiO_2_, in turn obtained by drying the gel in an oven for 24 h at 105 °C and grinding it in an agate mortar. The PeL material was obtained by mixing ZnO, Ga_2_O_3_, GeO_2_, and Cr_2_O_3_ in an agate mortar at a specific molar ratio (3:1:2) and then treated in the muffle furnace at 900 °C for 2 h and then at 1100 °C for additional 2 h. Eventually, ZnO was synthesized according to the nitrate-route, as previously described in Alberti et al. (2021a).

### Physico-chemical characterization

All physico-chemical characterization techniques exploited in the present work are reported in SI.

### Pilot photocatalytic plant

The pilot plant was studied and designed as tertiary module, to be put downstream of primary and secondary processes in WWTPs, specifically to face the problem of emerging pollutants. Different works reported in literature show different models of pilot plants (González-Pereyra et al. 2022; Merino-Mantilla et al. 2019); among the proposed ones, the most suitable was identified as the compound parabolic collector (CPC) solar photo-reactor (González-Pereyra et al. 2022; Sousa et al. 2012) which presents advantageous light-gathering properties and well-known design methodology (Luna-Sanguino et al. 2020). The prototype, shown in Figure S1, consists of a 1 L reactor (A), placed above a magnetic plate (B), and subjected to vigorous magnetic stirring. The container has an output connected to a pumping system (peristaltic pump, C), which allows the solution to flow continuously (max 1 L/min) in a glass coil (D), in which the solution can slide below the solar simulators. The coil, with a reduced diameter, is located above a tilting plane (E), covered in aluminum foil, and it ends up in the initial reservoir, to close the loop. This prototype cannot be exactly defined as CPC, but its design can be easily varied to adapt it, for example, changing the tilting plane below the glass coil. The solar radiation was simulated with two solar simulated light lamps (OSRAM ULTRA-Vitalux, 300W) placed at 20 cm above the coil.

### Chemometric approach (D-Optimal)

The study of heterogeneous photocatalysis as oxidation process for the abatement of emerging pollutants in a real plant is quite demanding; hence, it was decided to apply a chemometric approach to this study, selecting a proper mathematical model of experimental design compatible with numerous variables of both qualitative and quantitative nature. The design of choice was the D-Optimal, as it is the most suitable design, with respect to other models, to simultaneously manage a large number of variables (7) on a wide range of levels (which correspond to the different values of quantitative variables as well as different configurations of qualitative variables). The aim is to obtain information on the significance of each investigated variable and to evaluate any possible interactions occurring among variables. The D-Optimal model is described by a fundamental equation (Eq. 1), where the response *Y* is associated to each investigated variable (*X*_*n*_) and their respective significance coefficient (*b*_*n*_), together with the possible interactions terms (*X*_*n*_*X*_*m*_), and the respective interaction coefficients (*b*_*nm*_).

1 $$\begin{aligned}Y&={b}_{0}+{b}_{1}{X}_{1}+{b}_{2}{X}_{2}+{b}_{3}{X}_{3}+{b}_{4}{X}_{4}+{b}_{5}{X}_{5}+{b}_{6A}{X}_{6A}+{b}_{6B}{X}_{6B}+{b}_{7A}{X}_{7A}+{b}_{7B}{X}_{7B}+{b}_{7C}{X}_{7C}+{b}_{7D}{X}_{7D}+ {b}_{12}{X}_{1}{X}_{2}+{b}_{13}{X}_{1}{X}_{3}\\&+{b}_{14}{X}_{1}{X}_{4}+{b}_{15}{X}_{1}{X}_{5}+{b}_{23}{X}_{2}{X}_{3}+{b}_{24}{X}_{2}{X}_{4}+{b}_{25}{X}_{2}{X}_{5}+{b}_{34}{X}_{3}{X}_{4}+{b}_{35}{X}_{3}{X}_{5}+{b}_{45}{X}_{4}{X}_{5}+ {b}_{11}{X}_{1}^{2}+{b}_{22}{X}_{2}^{2}+{b}_{33}{X}_{3}^{2}\end{aligned}$$

The photocatalytic efficiency of the five synthesized samples was tested, in different experimental conditions, using MB, RhB, and MO as model pollutants, monitoring the decrease in absorbance by means of UV–Vis spectrophotometry as a function of time. The other variables monitored within this work were the pH of the solution, the concentration of the photocatalyst, the duration of the experiment, the position of the lamps, and the stirring speed of the solution.

The whole experimental domain investigated by the model is described by the chosen variables and the respective levels, reported in Table S1.

Experimental results are finally elaborated according to Eq. 1 using the coefficients obtained with a program able to compute the experimental design model (Chemometric Agile Tool; CAT) as reported elsewhere (Leardi et al. 2019). One of the main advantages in using this model is that the whole number of experiments is firstly calculated by CAT software, according to the defined experimental domain (considering all possible combinations of our investigated variables and their respective levels, the total number should be 1620). Having this set of candidate points, by the study the factors’ covariance, which indicates the degree of correlation between one variable and another, the mathematical model suggests then the minimum number of experiments required to get the most comprehensive information out of the experimental domain. This specific information is retrieved by studying the maximum inflation factor (MIF) as a function of the number of experiments, which should be equal to or less than 4, to be considered as acceptable (Benedetti et al. 2023). As an example, the factors’ covariance, represented with the MIF index, as a function of the number of experiments is reported in Fig. S2. In order to increase the covariance and thus decrease MIF to a value lower than 1, the final number of experiments was selected as 32. Experiments to be performed were randomly chosen by the program out of the 1620 possible experiments. Each experiment was performed to retrieve the final abatement degree of the model pollutant which is the response “Y” to be maximized in Eq. 1.

### Photodegradation experiments

All the experiments performed are carried out to retrieve the efficiency of the synthesized samples in different experimental conditions, as summarized in Table S1. The mathematical model helps to understand the significance of the chosen variables and possible interaction among variables which may enhance/decrease the efficiency. In order to do this, the dye solutions were subjected to photocatalytic tests and the abatement degree (Eq. 2) was calculated as a function of time of the experiment, followed by taking aliquots at fixed times, as follows:

2 $$Abb\mathrm{\%}\left(t\right)=\left[\frac{{C}_{0}-{C}_{t}}{{C}_{0}}\right]\times 100$$

where *Abb*%(*t*) represents the abatement degree for each time “*t*” corresponding to the time of aliquot withdrawal, calculated by means of the initial concentration “*C*_0_,” corresponding to 10 mg L^−1^, and the concentration of the dye along time “*C*_*t*_.” The dye concentration was calculated according to a calibration line performed prior to each experiment, thanks to the Beer-Lambert law. Quantitative measurements were performed with a Lambda 35 UV–Vis spectrophotometer (PerkinElmer), monitoring the absorbance at wavelengths of 664.6 nm, 464.4 nm, and 553.8 nm for MB, RhB, and MO, respectively.

### Recovery at pH_ZPC_

The possibility to quantitatively recover the suspended photocatalysts from the aqueous solutions treated in the proposed pilot plant was investigated by studying the point of zero charge of each sample. In fact, by adjusting the pH of the aqueous solution, it is possible to study the change in the Z-potential (*ζ*) value, which is related to the surface charge of the photocatalyst, to find the pH conditions for which the Z-potential becomes equal to 0. This is the condition known as point of zero charge, or isoelectric point, respectively reported as pH_PZC_ or pH_IEP_. *ζ* measurements were carried out using a Zeta sizer Nano ZS (Malvern Instruments Ltd, Malvern, UK). The measured electrophoretic mobilities were converted to *ζ*-potentials using Dispersion Technology Software (version 5.03, Malvern Instruments Ltd., UK). Once pH_ZPC_ was found, sedimentation experiments were carried out with a Turbidimeter (Turb 550, WTW, Germany) to measure the recovery rate. Samples were suspended in water (1 g/L), at the isoelectric point (pH_IEP_), and the sedimentation behavior of the samples was investigated as a function of time, by following the change in turbidity of the suspension, measure in nephelometric turbidity units (NTU).

## Results and discussion

### Characterization results

Different physico-chemical characterizations were employed to analyze the synthesized samples, to investigate their morpho-structural properties and define the correlation between the composite’s features and the displayed efficiency.

**Fig. 1:**
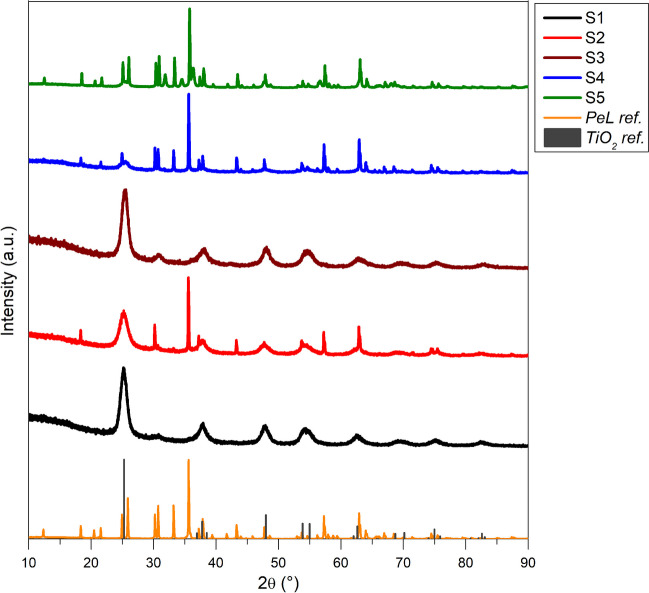
XRD normalized patterns of all samples and anatase TiO_2_ and PeL references

Figure 1 shows the XRD patterns obtained for all synthesized samples, together with anatase TiO_2_ reference pattern, retrieved from Pearson’s Crystal Data database, and bare PeL XRD pattern, used as reference for the PeL material.

The XRD analysis revealed the presence of the main phase of TiO_2_, i.e., anatase crystalline phase, visible in all samples. In particular, S1 (black line) shows only anatase phase peaks (as it is possible to assume by the comparison with the reference pattern (dark gray bars)) in the typical form of nanoparticles for the full width at half maximum of the peaks, attributable to the small dimension of the crystallites (also according to Scherrer equation, where the FWHM is inversely proportional to the crystallite’s size). S3 (wine line), analogously, shows diffraction peaks ascribable to the anatase phase, while an extra peak, centered at 30°, is the proof that the thermal treatment employed leads also to the formation of brookite as secondary phase (since only the main peak is visible in the pattern, it can be assumed that it is present in a very small amount). Supported samples, that are S2 (red line) and S4 (blue line), present both contributions of TiO_2_ and PeL to the patterns; at first sight, S4 seems to be slightly different in terms of weight percentage of each component, as S2 has more intense TiO_2_ peaks, while S4, conversely, has more intense PeL peaks. This must be attributed to the synthetic procedure employed. S5 (green line), which is a three-component supported sample, mainly shows the PeL pattern, as it is possible to notice by comparing with the PeL reference, while TiO_2_ presence is just touched upon. ZnO pattern (Alberti et al. 2021a) results instead to be not visible, mainly because it is also a component of the PeL material (as anticipated in the “Photocatalyst synthesis” section); thus, its presence in S5 sample is hidden by the PeL reference.

**Fig. 2:**
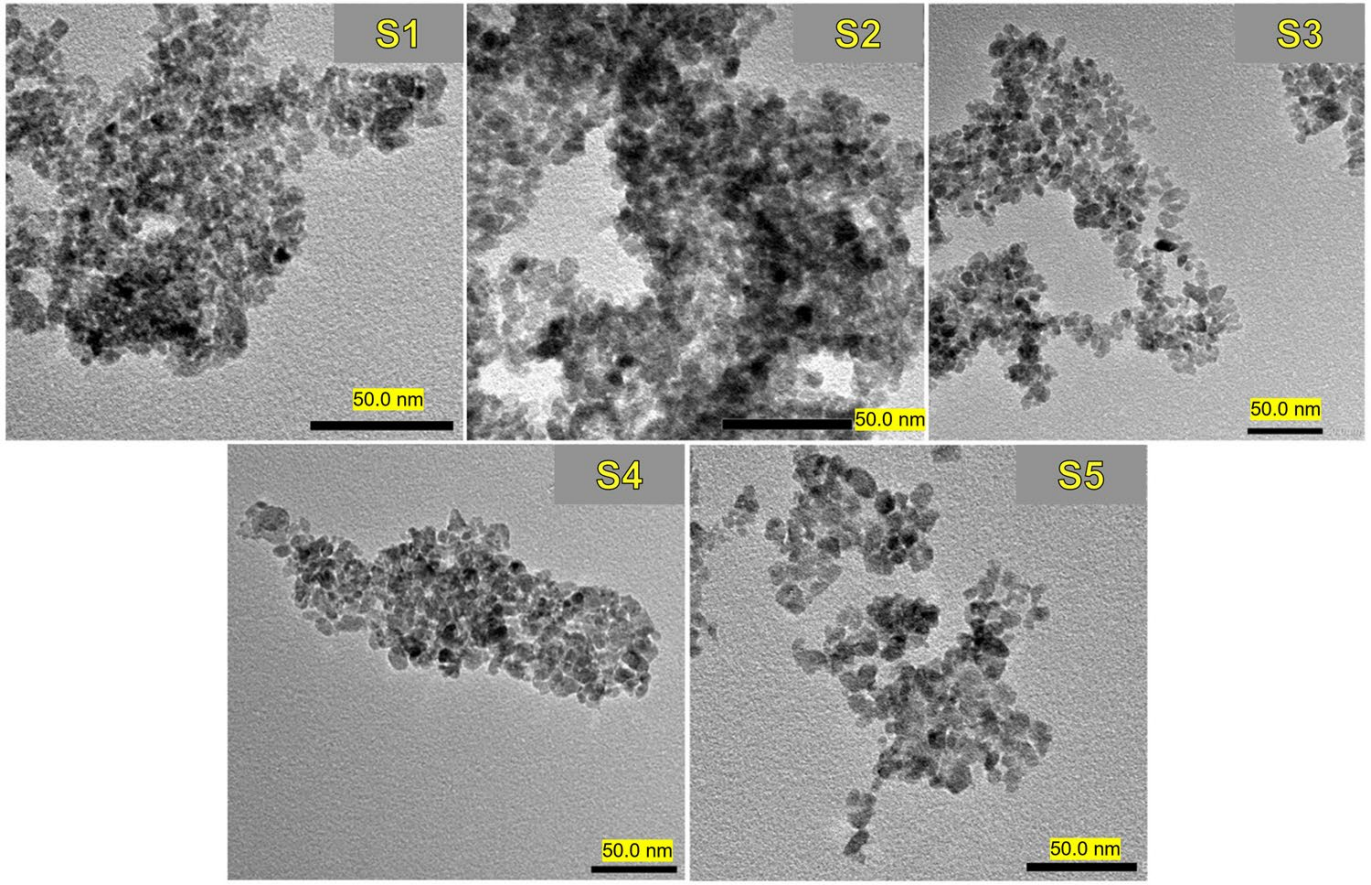
TEM images of the five different TiO_2_-based photocatalysts employed in the present work (for each image, the black marker is referred to 50 nm length)

The morphology of the synthesized TiO_2_ NPs at different temperatures with and without hydrothermal treatment was investigated via transmission electron microscope (TEM) as shown in Fig. 2. All samples revealed a semispherical irregular shape. The irregular morphology of TiO_2_ NPs was a consequence of the lack of steric stabilization exerted on the titanium cores before they agglomerate to form the TiO_2_ NPs. As the preparation temperature increased, the particle size slightly increased (with particle size from 5.6 to 13.5 nm from sample S1 to S5). The sample TiO_2_/ZnO/PeL (S5) revealed an improved dispersion compared to the other samples, and this is most likely attributed to the presence of PeL and solid-state treatment.

**Fig. 3:**
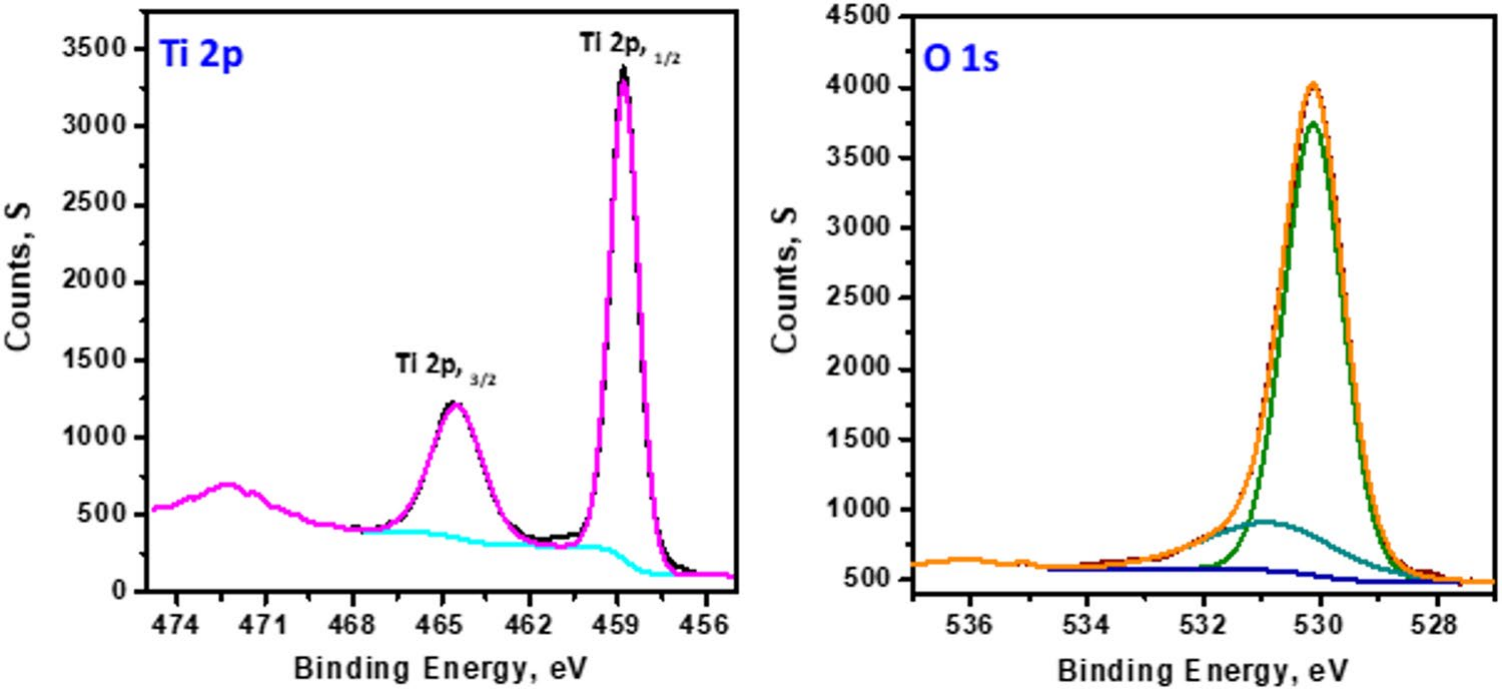
XPS of S1 sample, Ti 2p, and O 1 s spectra

To confirm the surface composition and oxidation state(s) of the prepared TiO_2_ NPs, X-ray photoelectron spectroscopic study (XPS) was performed and the results are shown in Fig. 3. The surface oxygen (O 1 s spectrum) is attributed to O^2−^ species in TiO_2_ (B.E. = 529.9 eV); a small peak appears at 531.2 eV and is assigned to OH − (surface hydroxyl) (Vasilopoulou 2014). The binding energies of Ti 2p_3/2_ and Ti 2p_1/2_ are observed approximately at 458.9 and 464.4 eV, respectively (Grbić et al. 2014). The ratio of the areas of the two peaks *A*(Ti 2p_1/2_)/*A*(Ti 2p_3/2_) is equal to 0.4, and the binding energy difference due to the spin–orbital coupling, Δ*E*_b_ = *E*_b_(Ti 2p_1/2_) − *E*_b_(Ti 2p_3/2_), was 5.7 eV in good agreement with the expected and reported value.

**Fig. 4:**
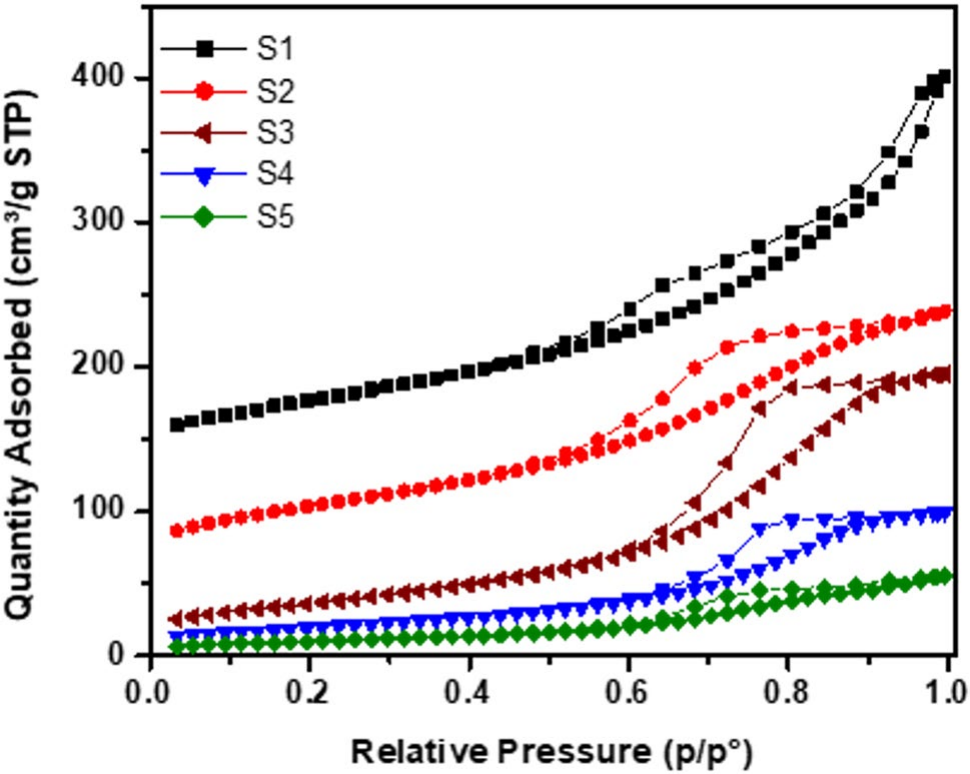
N_2_ adsorption isotherms of the five different TiO_2_-based photocatalysts employed

The porous structure of the prepared nanoparticles was investigated using nitrogen adsorption and desorption isotherms measured at 77 K, as shown in Fig. 4. All samples can be classed as type IV, which indicates the mesoporous nature of the prepared materials (as demonstrated in Table 2). For S1 sample, the adsorption isotherm can be classified as H3, whereas S2–S5 had a hysteresis shape between H1 and H3 (Thommes et al. 2015). The adsorption outlet S1 showed a significant increase at low pressure (< 0.01 *P*/*P*_0_), whereas S2–S5 exhibited a lower adsorption, the BET surface area of 189.22 m^2^ g^−1^ was obtained for S1 followed by a slightly higher surface area for S2 (195.00), and then, a decrease in surface area was measured at 131.09, 71.00, and 36.70 m^2^ g^−1^ for S3, S4, and S5, respectively. The high surface area values found for S1 and S2 can be safely attributed to the hydrothermal treatment employed during the synthesis; the presence of the PeL material did not affect this value. This could be due to the high oven temperature used for drying and the solid-state treatment at 350 °C.

**Table 2:** Pore size (nm), pore volume (cm^3^/g), and BET surface area of the NPs

Sample	Pore size (nm)	Pore volume (cm^3^/g)	BET surface area (m^2^/g)
S1	9.04	0.43	189.22
S2	5.98	0.29	195.00
S3	9.20	0.30	131.09
S4	8.70	0.15	71.00
S5	9.43	0.09	36.70

### Photodegradation experiments according to the D-Optimal chemometric approach

Once the number of experiments was selected to be 32, as described in the “Chemometric approach (D-Optimal)” section, photocatalytic tests were carried out according to the levels of each variable indicated by the model. Table S2 reports the experimental conditions selected for each performed experiments by the CAT software.

**Fig. 5:**
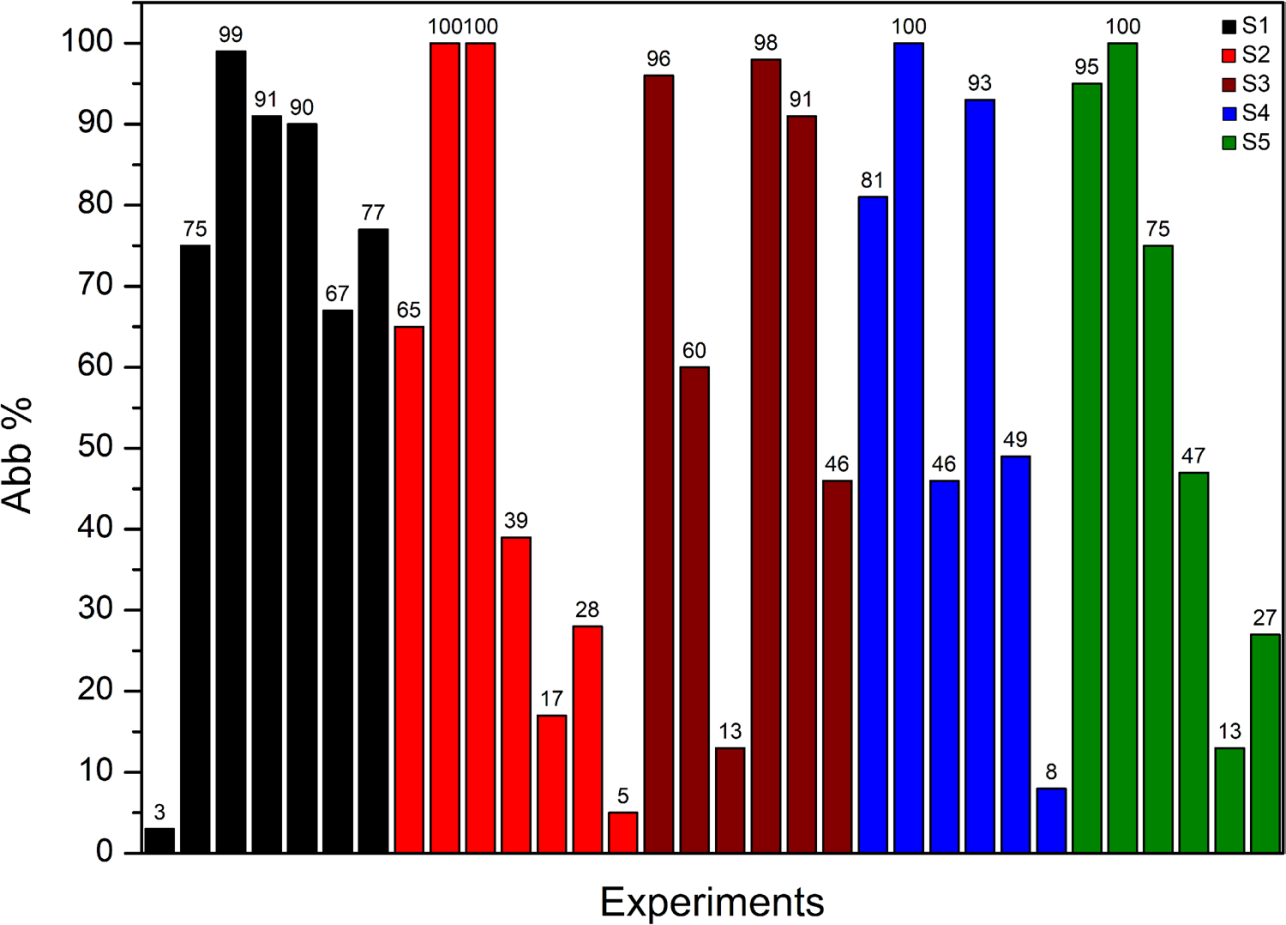
Experimental results obtained as Abb% degree for all performed experiments

Figure 5 shows the abatement degree calculated for all 32 experiments, in order to use the Abb% value as Y, obtained with the different *X*_*n*_, in Eq. 1. For clarity, it was decided to merge all experiments performed using the same photocatalyst by reporting them under the same color.

At this point, the model can be processed, and consequently, the significance graph, the values of the “*b*_*n*_” coefficients, and the response surfaces can be derived. By means of CAT program, once the final abatement values of each experiment have been entered, it is possible to solve the equation (Eq. 1) and assign a mathematical value to the “*b*_*n*_” coefficients, reported as a graph (Figure S3). Each coefficient is reported with an error bar, which defines the significance of each coefficient by reporting different *p* values, namely, *p < 0.05, **p < 0.01, and ***p < 0.001.

From the graph, it is possible to determine which experimental variables were found to be important and therefore necessary to be highlighted: only 4 out of 25 coefficients were mathematically significant, respectively related to the following variables: *X*_3_ (experiment duration), *X*_6a_ and *X*_6b_ (MB and RhB dyes), and *X*_3_ × *X*_4_ (interaction term between experiment duration and lamp position variables).

Consequently, the other coefficients and their related variables resulted to be not significant to increase the percentage abatement of the investigated pollutants; indeed, their absolute values were lower than the corresponding standard deviations. Focusing exclusively on the significant variables, we can see that the time (*X*_3_) has a positive coefficient, and this means that, within the experimental domain, a longer treatment time leads always to a higher percentage abatement. Therefore, increasing the treatment time when dealing with industrial pilot plants (designed as the one employed in this project) is a factor that must be considered. The other significant variables with a positive effect are *X*_6a_ and *X*_6b_, corresponding to the use of MB and RhB. Being qualitative variables, the information that we can retrieve with the model is that basically the photocatalytic samples can effectively interact and degrade these two specific pollutants, likely due to electrostatic interaction with samples’ surface or due to the molecular structure of the dye, which is easier to be degraded by photocatalytic reactions. This practically intends that some pollutants can be easily degraded in such pilot plant, while others can be more recalcitrant to photocatalytic degradation.

**Fig. 6:**
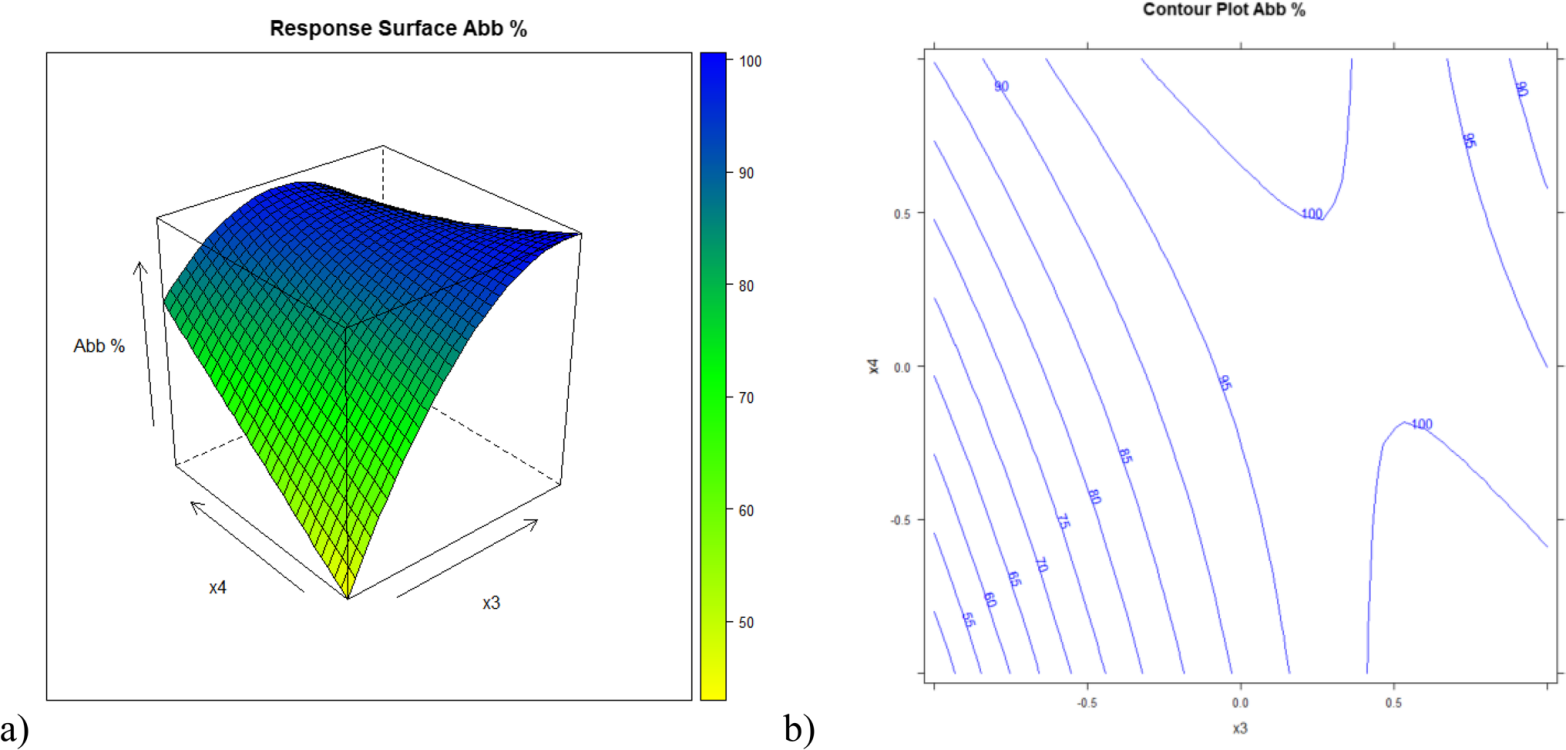
**a**) Response surface of *X*_*n*_ variables “lamp position *X*_4_ vs. experiment duration *X*_3_”; **b**) contour plot

Unexpectedly, the product *X*_3_ × *X*_4_ was also found to be significant, and for this reason, it was decided to report the response surface of these two variables to have a clearer vision (Fig. 6).

Figure 6a shows the relationship between time (*X*_3_) and lamp position (*X*_4_) variables, further underlined with the contour plot reported in Fig. 6b, which is a plane section of the response surface reporting the isolines of percentage abatement. The color scale on the right of Fig. 6a shows the response values (Abb%) ranging from 45% (yellow) to 100% (blue). It can be seen that a longer treatment time will lead to a higher dye degradation, as already mentioned above, but the additional information derived from the model is the interaction of this variable with the lamp positions, meaning that it is still also possible to obtain a high percentage of abatement with short time experiment simply by setting the solar lamps in a specific configuration (in our case, one lamp set above the coil and one above the container). This information can be thought of as practical hint for industrial photocatalytic plants, where time can be saved whether the irradiation with solar light is maximized in terms of exposed area.

### Material recovery

An important factor that must be addressed when dealing with photocatalytic pilot plant is the recovery of the suspended photocatalyst. In general, the recovery of nanoparticles can be challenging, as the possibility to use filters or centrifugation is strongly hindered by the small dimensions of the particles and the huge volume of solution that should be treated in pilot plants can impede the exploitation of such procedures.

For this reason, as preliminary attempt, a sedimentation test by gravity with the aid of an Imhoff cone was performed and subsequent recycle tests were carried out to investigate the reusability of the photocatalyst. MO degradation tests were performed with S1, repeated over seven consecutive cycles: S1 was dispersed in 500 mL of 20 mg L^−1^ MO aqueous solution and left treating for 5 h; at the end of the experiment, the whole dispersion was transferred to the Imhoff cone and was left settling down for 4 days. After the 4-day sedimentation, the supernatant was withdrawn leaving behind the smallest volume of solution (50–100 mL), still with the settled photocatalyst. This volume was then added to a fresh MO solution, to carry on with the recycling tests. It must be underlined that the sample was not collected, washed, and dried until the last cycle, where it was finally quantified by weighing it. Experimental results shown a 40% efficiency loss, passing from 98.7 to 60.2% at the end of the first and at the end of the seventh cycle, respectively. As anticipated, only after the end of the last cycle, the remaining solution was dried in an oven at 105 °C for 24 h to weigh the remaining amount of sample. As a result, only 50% of the initial sample was retained. Thus, it can be safely assumed that the efficiency loss can be due to sample loss, which proportionally lead to a decrease in the MO percentage abatement as the catalyst’s concentration decreased considerably (approximately 5% loss during each cycle). Furthermore, it is possible to suppose that the sample’s losses occurred between each consecutive cycle were due to the lack of control in the sedimentation tests, so that a part of the suspended sample did not undergo natural sedimentation and was then washed away with the supernatant.

In order to avoid the loss of nanoparticles and the subsequent loss in efficiency, it was crucial to investigate an innovative recovery technique for all samples. Prior to the recovery test, which gives insights into the possibility to safely employ powdered photocatalysts in water treatment plants, the pH_PZC_ was studied for all samples; results are shown in Table 3.

**Table 3:** pH_PZC_ values found for tested powder samples

Sample	pH_PZC_	Remarks
S1	5.77	-
S2	7.37	-
S3	8.19	-
S4	5.31	Sedimentation test performed at 6.29
	6.29	
	6.78	
S5	5.11	Sedimentation test performed at 5.98
	5.98	
	7.67	

We can see that some samples show more than one isoelectric point, and this is due to the existence in the supported samples of multiple materials other than titania showing different pH_PZC_ values.

The sedimentation behavior of the NPs was then recorded in terms of efficiency over time, as illustrated in Figure S4, and results are then summarized in Table 4.

**Table 4:** Recovery efficiencies (%) and time for complete sedimentation

Sample	Efficiency (%)	Time (× 10^3^ s)	Remarks
S1	97.6	88.7	Complete sedimentation after 1 day
S2	85.1	175	Complete sedimentation after 2 days
S3	97.3	88.7	Complete sedimentation after 1 day
S4	93.3	88.7	Complete sedimentation after 1 day
S5	99.3	88.7	Complete sedimentation after 1 day

Among tested samples, S5 resulted the fastest to be recovered at the highest efficiency, even though the same considerations may be done for all the other samples but S2. Results are promising, especially for the possibility of quantitative recovery of each sample at mild pH_PZC_ values. This outcome reflects how the exploitation of this recovery approach would highly enhance the retrieval of the photocatalysts used in water treatment plants and improve their safety, by impeding the loss of sample during multiple cycles.

## Conclusions

A multivariate chemometric approach was successfully applied to the case study of a scalable and home-made heterogeneous photocatalytic plant based on a CPC design, which is reported to be one the best design choices among others for big water treatment facilities (Grčić et al. 2024; Mustafa et al. 2023; Rapti et al. 2023). Particularly, the application of the D-Optimal design helped to dramatically reduce the number of experiments while simultaneously evaluating the significance of each variable as well as exploring the possible interaction between them, which would not be possible with a mono-variate approach. Owing to its application, it was possible to recognize that, in such processes, some variables are more significant than others (type of pollutant, experiment duration, and interaction between time and lamp positions); hence, during an industrial scale-up, they should be carefully monitored and controlled. It is to be noted that the literature reporting the application of a chemometric model for such purposes is very scarce so far (Ba-Abbad et al. 2017; Boutra et al. 2022; Deriase et al. 2021); thus, we can assume that its exploitation may help in dealing with the same research problems.

All synthesized samples were proved to be mainly composed of anatase phase of TiO_2_, even though S3 shown the presence of a small amount of secondary brookite, with dimensions lower than 10 nm, as disclosed by TEM. Surface area was recorded to be very high for the samples treated with hydrothermal process and with a higher isopropanol volume during sol–gel synthesis (S1 and S2). To the best of our knowledge, we can state that no composite materials relying on the coupling between a semiconductor and a luminescent material was ever studied in a photocatalytic plant, as recent reports only discuss about commercial TiO_2_ or general semiconductors fixed on solid supports (Chaubey et al. 2023; Grčić et al. 2024; Mustafa et al. 2023). Moreover, the sol–gel synthesis could be the right choice to maintain good levels of reproducibility, control over the purity, and morphology while scaling up towards a large-scale production. The hydrothermal heat-treatment should be also taken into consideration, but this would potentially become the bottleneck for the industrial production.

In addition, we focused on powdered composite materials thanks to the innovative method that we proposed for their recovery from aqueous suspensions. According to the most recent literature, there are some issues that should be still addressed while considering the practical application of photocatalysis on an industrial scale (Kuspanov et al. 2023), like the development of highly efficient photocatalysts with recycling possibility and minimal loss of photocatalytic activity. The immobilization of the photocatalyst on a solid base seems to be the most suitable option, but the reduction in exposed surface area is often proportional to the reduction of photoactivity. Hence, the possibility to recover the suspended NPs by reaching the isoelectric point would help in principle to avoid the need of solid supporting materials and let the whole exploitation of NPs’ potential.

S1 could be recycled for at least seven cycles, but the unattended recovery by sedimentation led to a decrease in efficiency of ca. 40%, which was imputed to the material loss. Additionally, the sedimentation took place for 4 days between each consecutive cycle. Among all samples, S2 resulted to be the slowest to be recovered while S5 the fastest. As the type of sample was not significant according to the chemometric outcome, it is possible to opt for the sample which is recovered faster, to further save time and decisively apply this kind of technology in actual treatment plants. The proposed approach was investigated to prove that the isoelectric point can allow the full recovery of the suspended photocatalysts, ensuring maximum efficiency in consecutive cycles.

Future developments may include the chemometric study of our synthesized photocatalysts in the pilot plant against real pollutants and in increasing treating volumes, carrying out a comprehensive study on the possible degradation by-products as well as the toxicity of the effluents. The service life of samples should also be precisely defined as well as the conduction of a complete cost estimation for this kind of systems for commercialization, which is still necessary.

### Supplementary Information

Below is the link to the electronic supplementary material.

Supplementary file1 (DOCX 2363 KB)

## Data Availability

Data available on request from the authors.
