# Supplementary material for: Synthesis and characterization of TiO2-based supported materials for industrial application and recovery in a pilot photocatalytic plant using chemometric approach
Source: Environ Sci Pollut Res Int. 2024 Feb 20;31(13):20556–67. doi: 10.1007/s11356-024-32467-y (PMC10927805; doi:10.1007/s11356-024-32467-y)
Supplement: Supplementary file 1 — Supplementary file1 (DOCX 2363 KB) [file 11356_2024_32467_MOESM1_ESM.docx]

**Synthesis and characterization of TiO_2_-based supported materials for industrial application and recovery in a pilot photocatalytic plant using chemometric approach**

**Nicolò Ghibaudo^1^, Maurizio Ferretti^1^, Entesar Al-Hetlani^2^, Metwally Madkour^3^, Mohamed O. Amin^2^ and Stefano Alberti^1, *^**

*^1^Chemistry and Industrial Chemistry Department, University of Genoa, via Dodecaneso 31, 16146, Genova (Ge), Italy.*

*^2^Chemistry Department, Faculty of Science, Kuwait University, P.O. Box 5969, Safat 13060, Kuwait.*

*^3^Chemistry Department, Faculty of Science, Arish University, Al-Arish 45511, Egypt.*

*Corresponding author’s e-mail: [stefano.alberti@unige.it](mailto:stefano.alberti@unige.it)

[nicolo.ghibaudo@edu.unige.it](mailto:nicolo.ghibaudo@edu.unige.it); [ferretti@chimica.unige.it](mailto:ferretti@chimica.unige.it); [entesar.alhetlani@ku.edu.kw](mailto:entesar.alhetlani@ku.edu.kw); [metwally.madkour@sci.aru.edu.eg](mailto:metwally.madkour@sci.aru.edu.eg); [mohamed.amin@ku.edu.kw](mailto:mohamed.amin@ku.edu.kw)

**Supporting Information**

**2.1 Chemicals**

Tetraisopropoxide Titanium (TTIP) (97%) and 2-propanol (i-PrOH) (99.9%) were purchased from Sigma-Aldrich (St. Louis, MO, USA), while distilled water was obtained with ARIOSO Water Purification System, (Human Corporation, Seoul, Republic of Korea). The PeL material was synthesized with zinc oxide ZnO (Alfa Aesar, 99.99%), gallium oxide Ga_2_O_3_ (Alfa Aesar, 99.999%), germanium oxide GeO_2_ (Alfa Aesar, 99.98%), and chromium oxide Cr_2_O_3_ (Alfa Aesar, 99.97%), as precursors, retrieved from Alfa Aesar (Kandel, Germany). ZnO employed for the S5 sample was synthesized using Zn(NO_3_)_2_*6H_2_O (98%, Sigma Aldrich, Darmstadt, Germany) and monohydrated citric acid (99.5+%, Alfa Aesar, Kandel, Germany). For the photocatalytic tests, Methylene Blue (MB) (Sigma Aldrich, Germany), Rhodamine B (RhB) (Alfa Aesar, Germany), and Methyl Orange (MO) (CARLO ERBA Reagents S.r.l., Cornaredo, Italy) were used.

**2.3 Physico-chemical characterization**

All samples were subjected to different physico-chemical characterization techniques, to address the composite materials’ features and correlate them to their efficiency. In particular, the following techniques were employed: X-Ray Diffraction “XRD” was performed for phase identification, with a 600 W Rigaku Miniflex (Bragg-Brentano, Cu Kα, Ni filtered, range 10–90 (2θ), step 0.01 (2θ), scan rate 5◦ min^-1^). Surface elemental analysis of the nanoparticles was performed using X-ray photoelectron spectroscopy (XPS) on an ESCALAB250 xi XPS spectrometer with an Al Kα monochromatic source and a charge neutralizer. Brunauer–Emmett–Teller (BET) method based on adsorption data in the relative pressure (P/P_0_) range of 0.02 to 0.22 using a model Gemini VII, ASAP 2020 automatic Micromeritics sorptometer algorithms. Prior to analysis, the samples were evacuated for 12 h at 110°C under a vacuum to remove any residual moisture. The morphology and particle size of the prepared NPs were examined using a JEM-3010 TEM (JEOL, Japan) operating at an accelerating voltage of 300 kV. The samples were dispersed in ethanol and dropped onto a carbon support film.


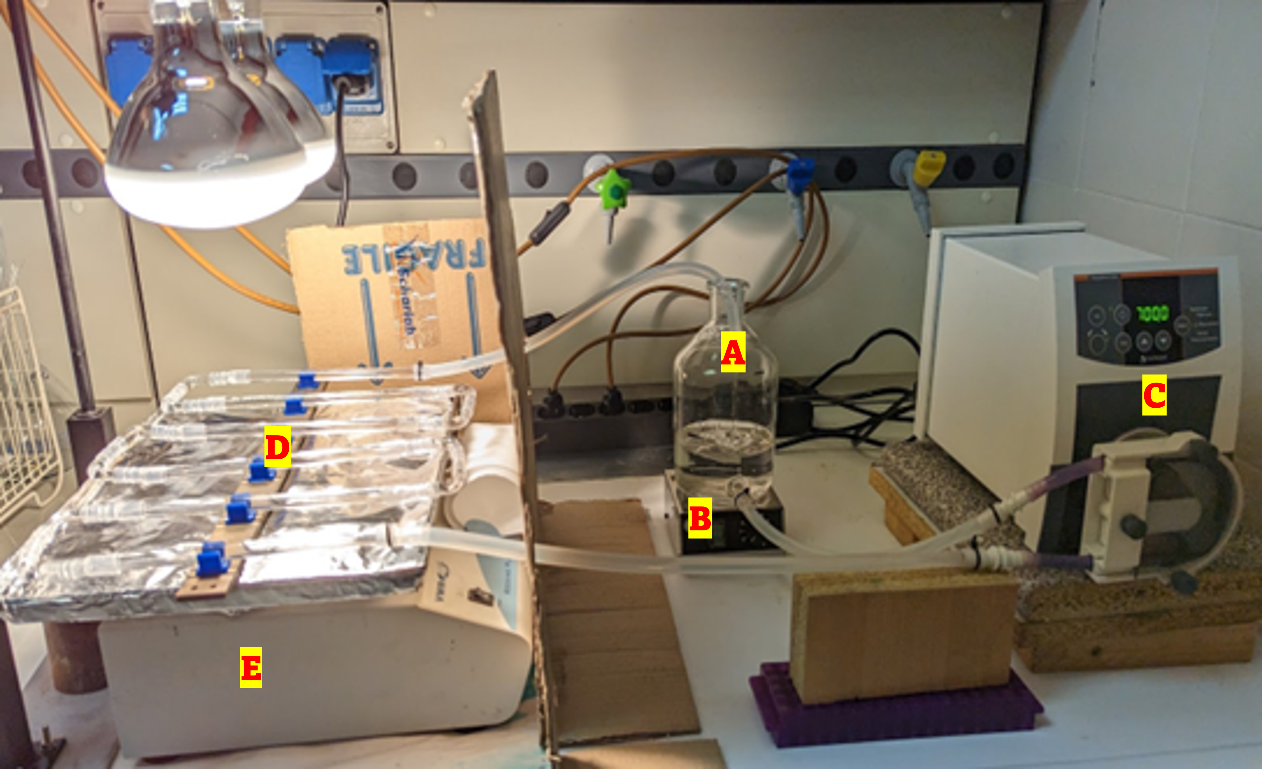


**Figure S1:** Prototype of pilot plant for water treatment used.

**Table S1**: Variables investigated in the D-Optimal model and their levels (experimental domain).

| *Variables* | | *Levels* |
| --- | --- | --- |
| X_1_ | Photocatalyst concentration | 0.5 g/L  1.0 g/L 1.5 g/L |
| X_2_ | pH | 5  7  9 |
| X_3_ | Experiment duration | 2 h  3,5 h  5 h |
| X_4_ | Lamps position | 2 lamps on coil  1 on coil 1 on container |
| X_5_ | Stirring speed | 200 rpm  800 rpm |
| X_6_ | Model Pollutant | MB  RhB  MO |
| X_7_ | Photocatalysts | S1  S2  S3  S4  S5 |


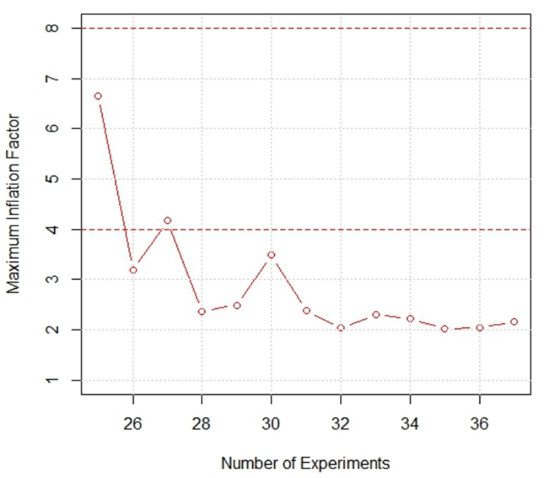


**Figure S2**: Dependance on the Maximum Inflation Factor upon the number of experiments to be performed.

**Table S2**: Experimental conditions of every photocatalytic test performed within the experimental domain.

| *Num. Experiment* | *X_1_* | *X_2_* | *X_3_* | *X_4_* | *X_5_* | *X_6_* | *X_7_* |
| --- | --- | --- | --- | --- | --- | --- | --- |
| *1* | 1,5 | 9 | 2 | 2 coil | 800 | MB | S1 |
| *2* | 0,5 | 5 | 5 | 2 coil | 800 | MB | S1 |
| *3* | 1,5 | 9 | 5 | coil + tank | 800 | RhB | S1 |
| *4* | 1,5 | 5 | 3,5 | coil + tank | 200 | RhB | S1 |
| *5* | 0,5 | 9 | 5 | coil + tank | 200 | RhB | S1 |
| *6* | 1,5 | 5 | 5 | 2 coil | 800 | MO | S1 |
| *7* | 1 | 9 | 5 | 2 coil | 200 | MO | S1 |
| *8* | 0,5 | 5 | 2 | 2 coil | 200 | MB | S2 |
| *9* | 1,5 | 7 | 5 | 2 coil | 200 | MB | S2 |
| *10* | 1,5 | 5 | 3,5 | 2 coil | 800 | RhB | S2 |
| *11* | 1 | 5 | 5 | coil + tank | 800 | RhB | S2 |
| *12* | 0,5 | 9 | 2 | 2 coil | 800 | MO | S2 |
| *13* | 1,5 | 5 | 2 | coil + tank | 800 | MO | S2 |
| *14* | 1,5 | 9 | 2 | coil + tank | 200 | MO | S2 |
| *15* | 0,5 | 5 | 3,5 | coil + tank | 800 | MB | S3 |
| *16* | 1,5 | 5 | 5 | coil + tank | 800 | MB | S3 |
| *17* | 1,5 | 5 | 2 | 2 coil | 200 | MB | S3 |
| *18* | 1,5 | 9 | 5 | 2 coil | 800 | RhB | S3 |
| *19* | 0,5 | 5 | 5 | 2 coil | 200 | RhB | S3 |
| *20* | 1 | 7 | 2 | coil + tank | 200 | MO | S3 |
| *21* | 0,5 | 9 | 2 | coil + tank | 800 | MB | S4 |
| *22* | 1,5 | 9 | 5 | coil + tank | 200 | MB | S4 |
| *23* | 1 | 7 | 2 | 2 coil | 800 | RhB | S4 |
| *24* | 0,5 | 5 | 2 | coil + tank | 200 | RhB | S4 |
| *25* | 0,5 | 7 | 5 | coil + tank | 800 | MO | S4 |
| *26* | 0,5 | 9 | 3,5 | 2 coil | 200 | MO | S4 |
| *27* | 0,5 | 9 | 5 | 2 coil | 800 | MB | S5 |
| *28* | 1 | 9 | 3,5 | coil + tank | 800 | MB | S5 |
| *29* | 1,5 | 7 | 2 | coil + tank | 800 | RhB | S5 |
| *30* | 1,5 | 9 | 2 | 2 coil | 200 | RhB | S5 |
| *31* | 0,5 | 5 | 2 | 2 coil | 800 | MO | S5 |
| *32* | 0,5 | 5 | 5 | coil + tank | 200 | MO | S5 |


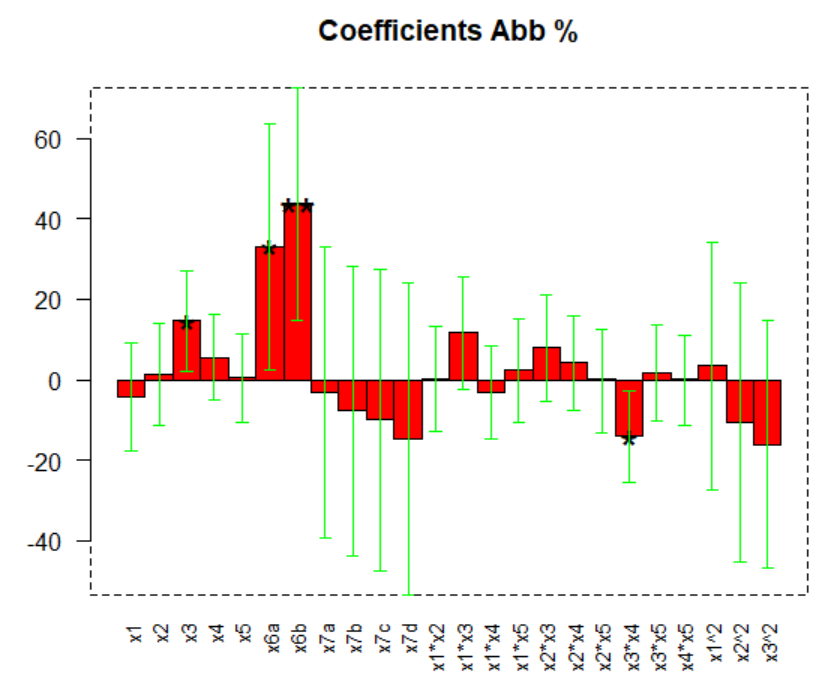


**Figure S3:** Coefficient values and their significance (*p<0.05, **p<0.01)


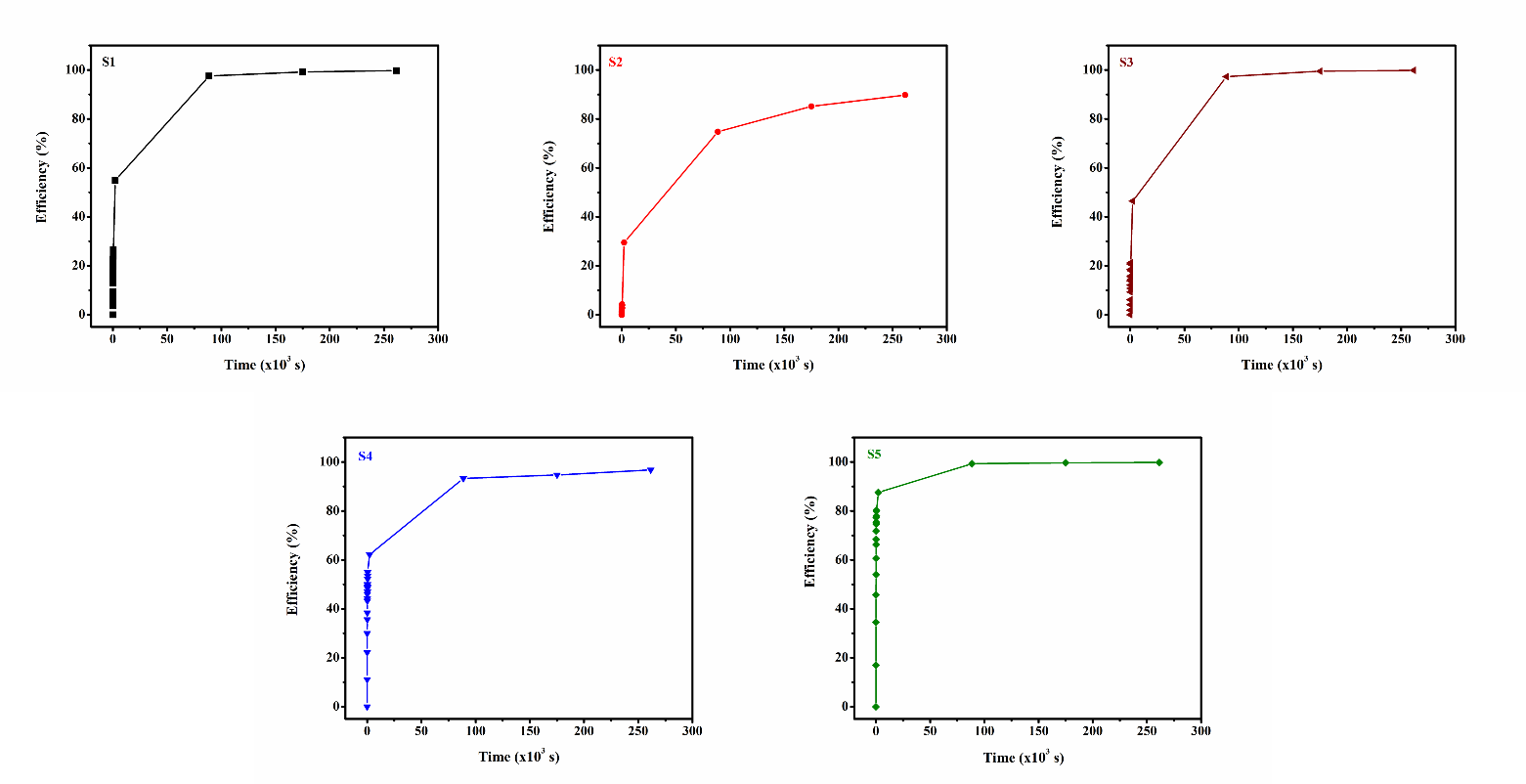


**Figure S4:** Sedimentation efficiency of each individual sample studied as function of time by means of turbidimetry.
